# Individual and regional characteristics associated with changes in mental health before and during the COVID-19 pandemic in South Korea

**DOI:** 10.1038/s41598-022-18493-1

**Published:** 2022-08-19

**Authors:** Jieun Min, Dohoon Kwon, Whanhee Lee, Cinoo Kang, Chaerin Park, Seulkee Heo, Michelle L. Bell, Ho Kim

**Affiliations:** 1 Department of Public Health Sciences, Graduate School of Public Health, Seoul National University, Seoul, Republic of Korea; 2 School of the Environment, Yale University, New Haven, CT USA; 3 Department of Biostatistics and Epidemiology, Graduate School of Public Health, Seoul National University, 1 Gwanak-ro, Gwanak-gu, Seoul, 151-742 Republic of Korea

**Keywords:** Epidemiology, Risk factors

## Abstract

Mental health has been a major public health concerns during the COVID-19 pandemic. This study investigated the effects of COVID-19 on mental health and whether individual and regional characteristics are associated with the changes in mental health. We estimated district-specific changes in the prevalence of moderate stress, extreme stress, and depression before and during the COVID-19 pandemic after adjusting for the time trend of mental health outcomes. Then, a meta-regression was conducted to examine the associations between district-level characteristics and changes in mental health due to the pandemic. The prevalence of moderate stress, extreme stress, and depression increased during the pandemic and the increases were more prominent in districts with high population density and in individuals aged 19–59 years, with a high education level, and with high household income. The % with reduced physical activity due to the pandemic were positively associated with increases in stress; while, the % that have mutual trust among neighbors and the number of sports facilities were negatively associated with increases in stress. Local tax per person had a positive association with increase in depression. Our study provides epidemiological evidence into the mental health consequences of the pandemic, which can inform the priority of resource allocation for managing mental health.

## Introduction

As of August 2021, the recorded number of confirmed cases of coronavirus disease 2019 (COVID-19) was more than 200 million worldwide, with more than 4 million deaths^1^. The spread of COVID-19 has affected not only the physical health of infected individuals^2,3^, but also the mental health and well-being of the general population^4^. Previous studies have suggested that mental health is one of the major public health concerns during the COVID-19 pandemic^4,5^ and the World Health Organization (WHO) announced that some public mental health service systems had collapsed during the pandemic^6^. An increase in psychological problems is associated with an increased risk of suicidal ideation and several chronic diseases^7–9^, and the resultant economic costs are expected to increase rapidly^10^; consequently, the ongoing COVID-19 pandemic is anticipated to increase the social and economic burden related to mental health^11^. Therefore, the effect of the COVID-19 pandemic on mental health status should be investigated.

Several studies have investigated the mental health during the COVID-19 pandemic through national surveys. The United Kingdom Household Longitudinal Study reported that the prevalence of mental health problems increased during the pandemic^4^. Three studies conducted in the United States revealed that the prevalence of mental disorders increased during the pandemic period, and the increase was pronounced in women, young adults, and low-income populations^12–14^. A Chinese study demonstrated that a reduction in emotional well-being was associated with the COVID-19 outbreak^15^. Nevertheless, most previous studies on the effect of the COVID-19 pandemic on mental health and well-being were mainly conducted in Western countries and China; thus, their results are limited in terms of generalization to other populations. Additionally, these studies were limited in reporting the underlying individual factors related to the effect of the pandemic on mental health, which can provide evidence to prioritize resources to address mental health concerns.

Mental health conditions can be affected by the characteristics of the region in which the individuals live. An increased proportion of green space is associated with a decrease in depression/anxiety levels owing to multiple factors such as increased physical activities, aesthetics of nature, and social cohesion^16^. A study conducted in Israel reported that residing in cities with high socioeconomic status (SES) was associated with higher mental illness than residing in cities with lower SES^17^. Moreover, residing in areas of high versus low population-density might impact levels of stress due to differences in crimes^18^, housing costs^19^, and accessibility of health care^20^. Despite these potential associations between mental health status and district-level characteristics, studies on the association of district-level characteristics with mental health changes due to the COVID-19 pandemic are scarce.

Therefore, this study aimed to investigate the changes in mental health conditions comparing periods before and during the COVID-19 pandemic period, and to examine individual and district-level characteristics associated with the changes in mental health, using a national community health survey conducted from 2017 to 2020.

## Materials and methods

### Study design

This research is pre-post comparison study on changes in mental health during the COVID-19 pandemic period (2020) compared to the previous three consecutive years (2017–2019), using the Korea Community Health Survey (KCHS) data. According to Article 2.2 of the Enforcement Rules of Bioethics and Safety Act of Korea, the KCHS is not included in Human Subjects Research. Therefore, the Korea Disease Control and Prevention Agency (KCDC) has announced that the Institutional Review Board review for KCHS has not been required since 2017. The KCHS obtained prior written consent from all participants and this survey is publicly available and completely de-identified.

In addition, the area of this study covered all of 229 districts (shi/gun/gu) in South Korea, and these districts indicate the second-level local authority districts comparable to ‘counties’ of the United States.

### Data

#### Korea Community Health Survey data

We analyzed KCHS data for all participants (total of 915,089 adults) living in the 229 districts from 2017 to 2020. The KCHS is a nationwide community-level health survey conducted annually by the KCDC since 2008. This survey aims to monitor various health indicators (e.g. prevalence of disease, smoking and drinking behaviors, and mental health) among communities and to establish community-specific and central public health plans^21^. The study participants were selected every year through probability proportionate sampling and systematic sampling from South Korean adults aged ≥ 19 years to represent the general adult population in South Korea.

#### Mental health variables

The primary mental health outcomes were the ‘subjective stress recognition status’ and ‘experience of depression’, based on two questions asked by the KCHS. For the subjective stress recognition status, we considered the question “How much stress do you feel in your daily life?” with available responses: ‘very much’, ‘a lot’, ‘a little bit’, and ‘rarely’. We categorized ‘subjective stress recognition status’ into ‘persons who recognized moderate stress’ as those who responded ‘very much’ or ‘a lot’ and ‘persons who did not recognized moderate stress’ as those who responded ‘a little bit’ or ‘rarely’. We further considered ‘persons who recognized extreme stress’ as participants who responded ‘very much’ to the same question, who are the subset of ‘persons who recognized moderate stress’. To assess the ‘experience of depression’, we considered the question “Have you ever felt depressed or despaired that might interfere with daily life in a recent year?”, which had two available responses: ‘yes’ and ‘no’. Then, ‘participants with depression’ were defined as individuals who responded ‘yes’ to the depression experience question.

#### Confounders

To consider the potential confounding effects, we used factors related to demographics (sex, age, subjective health level, smoking status, and drinking status), SES (education level, employment status, household income, marital status, and currently living alone), and chronic disease history (hypertension and diabetes). Detailed information on these variables and their categorizations are described at Supplementary Table S1.

#### District-level variables

We collected seven district-level variables reflecting SES (local tax per person), social isolation (% that have mutual trust among neighbors), greenspace (park area per capita), recreational activities (# of sports facilities per 100,000 people), and pandemic-related variables (% with reduced physical activity due to the pandemic, % who believe in the government responses to the pandemic, and # of COVID-19 confirmed cases). The definitions and data sources of these variables are presented in Supplementary Table S2.

### Statistical analyses

To estimate changes in mental health outcomes before and during the COVID-19 pandemic and to determine if regional characteristics are associated with these changes, we performed a two-stage analysis. We conducted all statistical analyses using R statistical software, version 4.1.0.

In the first stage, the logistic regression considering the survey structure was applied to data from each district separately to estimate the district-specific time-trend adjusted change in mental health comparing the period of the pandemic to the timeframe before the pandemic. A binary variable indicating the pre- or post-outbreak of the pandemic was included in the logistic model to quantify district-specific changes in mental health outcomes between 2017 and 2019 (pre) and 2020 (post). The year variable was adjusted linearly to control for the temporal trend. All confounders were adjusted for in the first-stage model.

In the second stage, we pooled the 229 district estimates to estimate the overall change in mental health outcomes during the pandemic, which was expressed as odds ratio (OR; OR > 1 indicates an increase in outcome in 2020 compared with that in the previous 3 years), separately for each health outcome. A random-intercept meta-regression model was used to examine the district-level characteristics associated with changes in mental health outcomes. The estimated association between district-level characteristics and changes in mental health outcomes was expressed as the change in OR of mental health outcome per interquartile range (IQR) increase of district-level variables. We adjusted for longitude and latitude to mitigate the unmeasured spatial heterogeneities. Because the correlations among the seven district-level variables were substantially high, we performed a separate meta-regression analysis for each district-level variable, based on previous studies that applied meta-regression models with highly correlated explanatory variables^22,23^. In addition, to identify factors that best explain the co-variability of the district-level variables, we conducted a principal component analysis (PCA)^23^. As shown in a score plot and a loading plot, the principal components showed groups of indicators (Supplementary Fig. S1).

#### Sub-district and sub-population analyses

To examine the different patterns of changes in mental health according to the regional and individual characteristics, we performed sub-district and sub-population analyses by repeating the two-stage analysis for each sub-district and sub-population. Details of these analyses are described in Supplementary methods online.

#### Sensitivity analysis

As a sensitivity analysis, we performed multiple regression analysis using district-specific age-standardized rate changes of mental health prevalence as response variables, instead of using the estimated changes in mental health outcomes calculated from the main analysis as response variables.

### Ethics approval

Not required. Korea Community Health Survey (KCHS) is not be included in Human Subjects Research, and the KCHS data is publicly accessible and completely anonymous without any personal information. In addition, the entire statistical analyses in this study were performed with the publicly available and de-identified KCHS data provided by the Korea Disease Control and Prevention Agency.

## Results

Table 1 summarizes the number of people who experienced moderate stress, extreme stress, and depression before (2017–2019) and during (2020) the COVID-19 pandemic by covariates. Among the total 915,089 study participants, 209,071 (22.8%), 28,545 (3.1%), and 54,426 (5.9%) experienced moderate stress, extreme stress, and depression, respectively. The proportion of individuals aged 19–59 years among individuals who experienced each mental health problem increased in the year 2020. Supplementary Tables S3–S4 present top 20 districts with low and high prevalence of moderate stress, extreme stress, and depression before the outbreak of the pandemic and the changes in prevalence of mental health outcomes during and before the pandemic. Table 2 shows the median values of district-level variables by population density.

**Table 1:** Baseline characteristics of study participants according to mental health before (2017–2019) and during (2020) the COVID-19 pandemic.

	Moderate stress, case (%)		Extreme stress, case (%)		Depression, case (%)	
	2017–2019^a^ (n = 685,820)	2020 (n = 229,269)	2017–2019^a^ (n = 685,820)	2020 (n = 229,269)	2017–2019^a^ (n = 685,820)	2020 (n = 229,269)
Total	158,342	50,729	21,654	6891	41,587	12,839
**Sex**						
Female	90,399 (57.1)	28,663 (56.5)	12,363 (57.1)	3857 (56.0)	28,486 (68.5)	8655 (67.4)
Male	67,943 (42.9)	22,066 (43.5)	9291 (42.9)	3034 (44.0)	13,101 (31.5)	4184 (32.6)
**Age**						
19–59 years	107,768 (68.1)	36,115 (71.2)	14,674 (67.8)	5,158 (74.9)	22,511 (54.1)	7501 (58.4)
60 + year	50,574 (31.9)	14,614 (28.8)	6980 (32.2)	1733 (25.1)	19,075 (45.9)	5338 (41.6)
**Subjective health level**						
Bad	47,804 (30.2)	9959 (19.6)	9117 (42.1)	1969 (28.6)	18,769 (45.1)	4056 (31.6)
Normal	69,794 (44.1)	21,228 (41.8)	8209 (37.9)	2746 (39.8)	15,867 (38.2)	5110 (39.8)
Good	40,722 (25.7)	19,540 (38.5)	4325 (20.0)	2175 (31.6)	7124 (17.1)	3672 (28.6)
Non-response	22 (0.0)	2 (0.0)	3 (0.0)	1 (0.0)	7 (0.0)	1 (0.0)
**Smoking status**						
Never-smoker	97,805 (61.8)	31,680 (62.4)	12,477 (57.6)	3925 (57.0)	27,702 (66.6)	8515 (66.3)
Past-smoker	25,295 (16.0)	8203 (16.2)	3364 (15.5)	1114 (16.2)	6424 (15.4)	1995 (15.5)
Current-smoker	35,230 (22.2)	10,843 (21.4)	5810 (26.8)	1852 (26.9)	7458 (17.9)	2328 (18.1)
Non-response	12 (0.0)	3 (0.0)	3 (0.0)	0 (0.0)	3 (0.0)	1 (0.0)
**Drinking status**						
Never-drinker	24,337 (15.4)	9789 (19.3)	3509 (16.2)	1228 (17.8)	8101 (19.5)	3093 (24.1)
Past-drinker	23,827 (15.0)	8516 (16.8)	3588 (16.6)	1189 (17.3)	8626 (20.7)	2729 (21.3)
Current-drinker	110,168 (69.6)	32,419 (63.9)	14,555 (67.2)	4474 (64.9)	24,855 (59.8)	7017 (54.7)
Non-response	10 (0.0)	5 (0.0)	2 (0.0)	0 (0.0)	5 (0.0)	0 (0.0)
**Education level**						
Less than college	93,296 (58.9)	27,851 (54.9)	13,002 (60.0)	3691 (53.6)	29,765 (71.6)	8514 (66.3)
College or higher	64,820 (40.9)	22,802 (44.9)	8610 (39.8)	3191 (46.3)	11,756 (28.3)	4313 (33.6)
Non-response	226 (0.1)	76 (0.1)	42 (0.2)	9 (0.1)	66 (0.2)	12 (0.1)
**Employed**						
No	53,345 (33.7)	17,339 (34.2)	8122 (37.5)	2474 (35.9)	21,870 (52.6)	6663 (51.9)
Yes	104,950 (66.3)	33,377 (65.8)	13,525 (62.5)	4416 (64.1)	19,695 (47.4)	6171 (48.1)
Non-response	47 (0.0)	13 (0.0)	7 (0.0)	1 (0.0)	22 (0.1)	5 (0.0)
**Household income**^**b**^						
Low-income	72,509 (45.8)	21,415 (42.2)	10,826 (50.0)	3079 (44.7)	25,630 (61.6)	7477 (58.2)
High-income	82,674 (52.2)	28,827 (56.8)	10,370 (47.9)	3737 (54.2)	15,076 (36.3)	5225 (40.7)
Non-response	3159 (2.0)	487 (1.0)	458 (2.1)	75 (1.1)	881 (2.1)	137 (1.1)
**Marital status**						
Married	103,200 (65.2)	31,328 (61.8)	16,420 (75.8)	4083 (59.3)	23,154 (55.7)	6744 (52.5)
Divorced	8145 (5.1)	3029 (6.0)	1396 (6.4)	477 (6.9)	3324 (8.0)	1157 (9.0)
Widowed	15,035 (9.5)	3912 (7.7)	2209 (10.2)	482 (7.0)	7965 (19.2)	2071 (16.1)
Separated	2408 (1.5)	1554 (3.1)	372 (1.7)	213 (3.1)	886 (2.1)	476 (3.7)
Never married	29,354 (18.5)	10,874 (21.4)	4221 (19.5)	1626 (23.6)	6192 (14.9)	2383 (18.6)
Non-response	200 (0.1)	32 (0.1)	36 (0.2)	10 (0.1)	66 (0.2)	8 (0.1)
**Currently living alone**						
No	135,268 (85.4)	43,458 (85.7)	18,108 (83.6)	5816 (84.4)	31,542 (75.8)	9794 (76.3)
Yes	23,074 (14.6)	7271 (14.3)	3546 (16.4)	1075 (15.6)	10,045 (24.2)	3045 (23.7)
**Hypertension history**						
No	117,851 (74.4)	38,685 (76.3)	15,821 (73.1)	5277 (76.6)	27,871 (67.0)	8995 (70.1)
Yes	40,440 (25.5)	12,037 (23.7)	5823 (26.9)	1612 (23.4)	13,689 (32.9)	3841 (29.9)
Non-response	51 (0.0)	7 (0.0)	10 (0.0)	2 (0.0)	27 (0.1)	3 (0.0)
**Diabetes history**						
No	140,725 (88.9)	45,264 (89.3)	18,927 (87.4)	6123 (88.9)	35,390 (85.1)	11,040 (86.0)
Yes	17,564 (11.1)	5458 (10.8)	2716 (12.5)	766 (11.1)	6170 (14.8)	1797 (14.0)
Non-response	53 (0.0)	7 (0.0)	11 (0.1)	2 (0.0)	27 (0.1)	2 (0.0)

^a^The number of cases in 2017–2019 are sum of the number of cases in 2017, 2018, and 2019.

^b^Low-household income corresponds to household income less than 3 million won (about 2600$) per month, and high-household income corresponds to household income more than 3 million won (about 2600$) per month.

**Table 2:** Descriptive statistics of district-level characteristics by population density.

Variable	Median (Q1, Q3)			
	Overall (229 districts)	Low population-density^a^ (76 districts)	Mid population-density^a^ (76 districts)	High population-density^a^ (77 districts)
Local tax per person (100,000 won)	11.0 (8.4, 14.9)	9.2 (7.6, 12.3)	13.2 (10.5, 16.7)	10.5 (8.2, 14.9)
% that have mutual trust among neighbors	62.4 (54.8, 71.7)	74.4 (69.7, 80.7)	61.3 (55.6, 65.5)	54.4 (48.5, 59.5)
Park area per capita (m^2^)	15.8 (7.4, 25.0)	23.2 (15.5, 32.4)	18.3 (13.1, 27.3)	6.9 (3.2, 12.0)
# of sports facilities per 100,000 people	3.27 (0.84, 9.13)	11.0 (7.4, 15.1)	3.7 (2.6, 6.6)	0.7 (0.4, 1.1)
% with reduced physical activity due to the pandemic	50.5 (42.3, 56.3)	40.4 (33.3, 46.8)	51.16 (46.0, 56.1)	56.0 (53.6, 58.9)
% that believe in the government responses to the pandemic	69.0 (63.7, 73.8)	72.1 (63.7, 77.9)	68.4 (62.8, 72.5)	68.6 (64.8, 71.2)
# of COVID-19 confirmed cases	541 (279, 5403)	279 (185, 667)	526 (189, 1587)	5403 (592, 6011)

*Q1* first quartile, *Q3* third quartile.

^a^Three sub-districts (low-density, mid-density, and high-density districts) were divided based on 33.3% and 66.7% percentile of population density.

**Figure 1:**
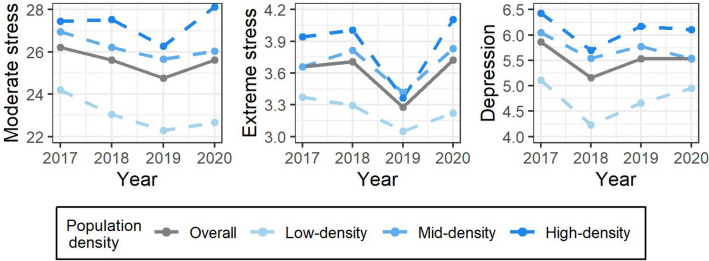
Time-series plot for standardized rate of mental health prevalence by population density during 2017–2020. The mental health prevalence was standardized by age based on the 2005 census.

Figure 1 shows the time trends of the age-standardized prevalence of mental health outcomes by population density from 2017 to 2020. The prevalence of mental health outcomes increased in the overall study area in 2020 compared with the average during 2017–2019. Districts with high population-density tended to have a higher prevalence of mental health outcomes than compared with districts with mid and low population-density during the overall study period.

**Figure 2:**
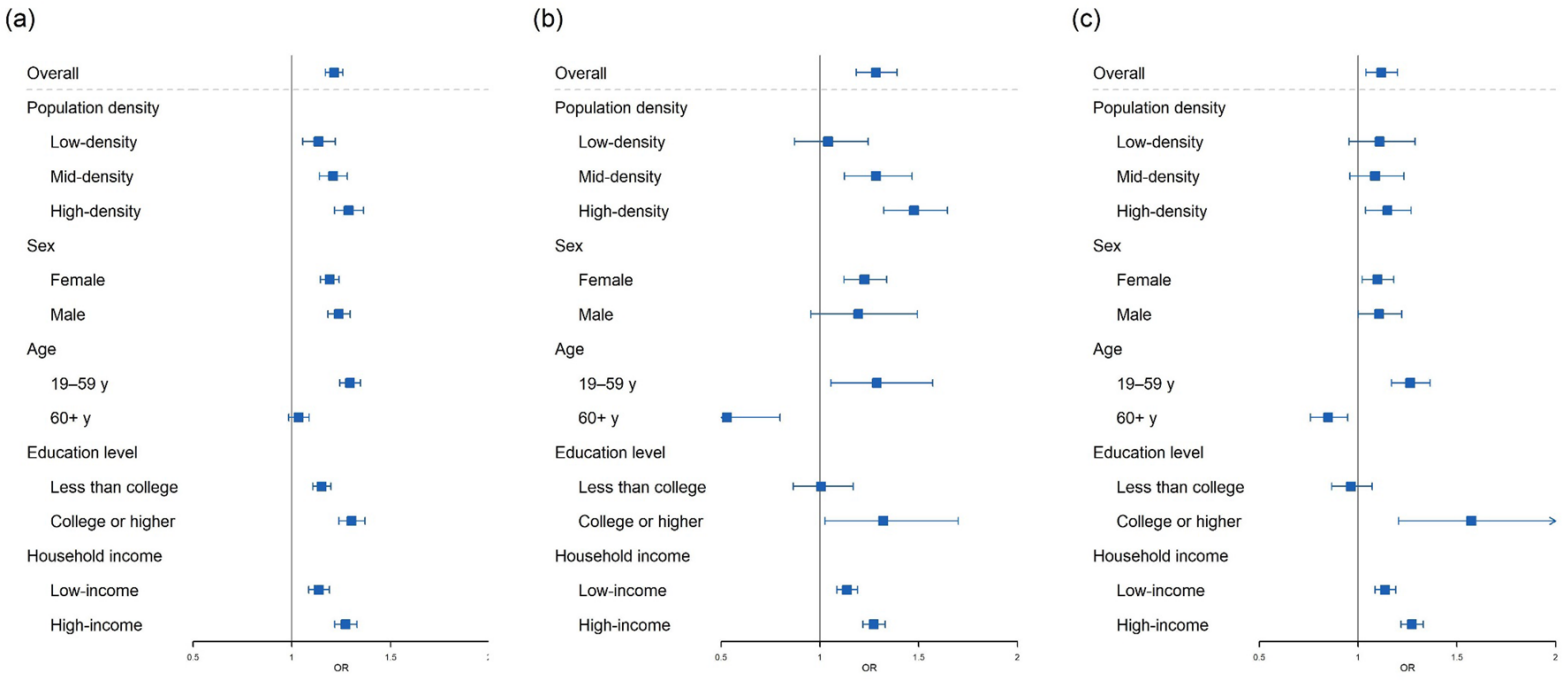
Time-trend adjusted OR of mental health by the outbreak of the COVID-19 pandemic according to sub-district and sub-population. (**a**) Moderate stress; (**b**) Extreme stress; and (**c**) Depression. ORs and 95% CIs were calculated from pooled-analysis after adjusting for time trend and individual characteristics. Low-household income corresponds to household income less than 3 million won (about 2600$) per month, and high-household income corresponds to household income more than 3 million won (about 2600$) per month. *CI* confidence interval, *OR* odds ratio.

Figure 2 presents the pooled time-trend adjusted changes in mental health outcomes due to the pandemic. During the pandemic, the prevalence of moderate stress, extreme stress, and depression increased with ORs 1.21 (95% CI 1.17, 1.26), 1.28 (95% CI 1.18, 1.39), and 1.12 (95% CI 1.04, 1.20), respectively. Furthermore, high population-density districts showed higher ORs than mid and low population-density districts. This result is shown geographically in Supplementary Fig. S2. Individuals who were aged 19–59 years, with a high education level, and with high household income showed higher increases of prevalence in all mental health outcomes than those who were aged 60 + years, with a low education level, and low household income, respectively. For moderate stress, the ORs in participants aged 19–59 years did not show evident differences depending on their household income, while the OR in participants aged 60 + years with higher household income was greater than that in participants with lower household income (Supplementary Fig. S3). Finally, there were no evident differences in the ORs between female and male individuals across all mental outcomes.

Table 3 displays the associations between district-level characteristics and changes in mental health outcome (i.e. estimated ORs from the two-stage analysis) before and during the pandemic. For moderate stress, the % that have mutual trust among neighbors and the number of sports facilities per 100,000 people showed a negative association with the increases in moderate stress; the decreases in ORs per IQR increase were 5.9% (95% CI − 10.8, − 0.7) and 7.4% (95% CI − 11.4, − 3.2), respectively. While, the % with reduced physical activity due to the pandemic was positively associated with the increase in moderate stress; the increases in ORs per interquartile range (IQR) increase was 10.6% (95% CI 5.4, 16.0). For extreme stress, the associations between district-level characteristics and changes in extreme stress showed the same pattern as that with moderate stress. For depression, local tax per person has a positive association with increase in depression; the increase in OR per IQR increase was 5.7% (95% CI 1.4, 10.3).

**Table 3:** Associations between district-level characteristics and changes in mental health comparing the period of the COVID-19 pandemic (2020) to before the pandemic (2017–2019).

	Percentile change in OR (95% CI)^a^
**Moderate stress**	
Local tax per person (100,000 won)	0.9 (− 1.1, 3.0)
% that have mutual trust among neighbors	− 5.9 (− 10.8, − 0.7)*
Park area per capita (m^2^)	− 3.1 (− 6.4, 0.3)
# of sports facilities per 100,000 people	− 7.4 (− 11.4, − 3.2)*
% with reduced physical activity due to the pandemic	10.6 (5.4, 16.0)*
% that believe in the government responses to the pandemic	− 0.3 (− 6.0, 5.8)
# of COVID-19 confirmed cases	6.4 (− 2.6, 16.4)
**Extreme stress**	
Local tax per person (100,000 won)	1.4 (− 2.9, 5.8)
% that have mutual trust among neighbors	− 18.3 (− 27.6, − 7.8)*
Park area per capita (m^2^)	− 0.5 (− 7.8, 7.5)
# of sports facilities per 100,000 people	− 14.3 (− 22.4, − 5.4)*
% with reduced physical activity due to the pandemic	24.4 (11.1, 39.2)*
% that believe in the government responses to the pandemic	− 5.7 (− 17.6, 8.0)
# of COVID-19 confirmed cases	10.7 (− 9.6, 35.4)
**Depression**	
Local tax per person (100,000 won)	5.7 (1.4, 10.3)*
% that have mutual trust among neighbors	− 1.6 (− 11.8, 9.9)
Park area per capita (m^2^)	0.3 (− 6.4, 7.4)
# of sports facilities per 100,000 people	3.6 (− 5.2, 13.1)
% with reduced physical activity due to the pandemic	1.5 (− 8.3, 12.3)
% that believe in the government responses to the pandemic	− 10.3 (− 20.4, 1.1)
# of COVID-19 confirmed cases	7.4 (− 10.3, 28.7)

*CI* confidence interval, *OR* odds ratio.

*p < 0.05.

^a^Percentile changes in ORs and 95% CIs were calculated from meta-regression after adjusting for longitude and latitude of each district. Results were expressed as percentile change in OR of mental health for interquartile range (IQR) increase of the district-level variables.

Results from sub-district and sub-population analyses corresponding to Table 3 are presented in Supplementary Tables S5–S7. The association between district-level characteristics and changes in moderate stress was more prominent in women and individuals with low education levels than men and those with high education levels, individually.

Sensitivity analysis revealed that our results were consistent even if we changed our main outcome variables to observe the standardized rate change of mental health prevalence (Supplementary Table S8).

## Discussion

We investigated the impact of COVID-19 pandemic on mental health outcomes, using a nationwide community health survey in South Korea. Among the total population, we found that the prevalence of moderate stress, extreme stress, and depression increased during the pandemic period (2020) after adjusting for the temporal trend of mental health, and increasing patterns were more pronounced in high population-density districts, and individuals who were aged 19–59 years, those with high education levels, and those with high household income than in mid and low population-density districts and in the general population. Also, the higher increases in moderate and extreme stress were associated with lower % that have mutual trust among neighbors; while, higher number of sports facilities and lower % with reduced physical activity due to the pandemic were associated with less increased stress during the pandemic. Furthermore, districts with higher local tax per person showed a higher increase in depression.

During the pandemic, the prevalence of moderate stress, extreme stress, and depression increased by 21%, 28%, and 12%, respectively. Our findings were generally consistent with those of previous studies^4,5,12–15^. Studies conducted in the United States reported that the prevalence of moderate and severe depression increased by nearly threefold during the pandemic, and increases in psychological problems were higher in younger adults than older adults by more than 10%^12,14^. A study conducted in the United Kingdom showed an increase in mental health prevalence by more than 5.5% among individuals with high education levels and high household income compared with individuals low education levels and low household income, respectively^4,5^. Another study conducted in South Korea also reported that the prevalence of depression increased by 49.8% during the pandemic, and the scores of moderate- to high-intensity exercise and social relationships decreased by 30% and 10% compared with those before the COVID-19 pandemic, respectively^24^. Other Chinese studies reported that the prevalence of mental illness in urban areas was 15.3%, which was significantly higher than 12.5% in rural areas during the pandemic^25^.

There are several plausible mechanisms that can explain the degenerated mental health during the pandemic. First, strong social distancing measures could affect the increase in mental health problems. Second, exposure to stressful news could indirectly affect the increase in psychological problems^4^. Third, social isolation, due to the fear of infection and restriction of social contact, could directly affect the increase in stress levels^26^. Fourth, a decrease in physical activities during the pandemic could also have a negative effect on mental health^27^. Finally, early compliance with social distancing policies could affect the increase in the prevalence of depression.

The increasing pattern in the prevalence of mental health outcomes during the pandemic differed by sub-district divided by population density, and the increase was more evident in high population-density districts than in other districts. We postulated that the more pronounced increase in prevalence of mental health outcomes in high population-density districts could be related to stricter restrictions applied to the same area. During the pandemic, the number of confirmed COVID-19 cases was the highest in high-population density districts (see Table 2); thus, restrictions in social gatherings were stronger in metropolitan/urban areas than in small urban and rural areas in South Korea^28^. Thus, in urban areas, the number of people allowed to enter restaurants was regulated, and public transportation operations were also decreased. Although these restrictions might be effective in reducing the spread of COVID-19^29^, they could cause the inconvenience and impose additional stress to urban residents. Our findings provide epidemiological evidence for mental health policies that are more prioritized for urban populations.

Our study also showed that younger people and individuals with a high SES showed more evident increase in mental health prevalence during the pandemic. We conjecture that the disparities in accessibility to information could be majorly associated with these results. Previous studies reported that individuals confront anxiety-provoking information through the social media, and more frequent exposures to such information can cause higher vulnerability to mental health degeneration when the future is unpredictable^30^. In addition, young and middle-aged, highly educated, and higher income populations may be more likely exposed to negative information from the Internet and social media than the general population^31,32^. Therefore, this might be related to a higher increase in the prevalence of mental health outcomes during the pandemic.

In this study, a lower % that have mutual trust among neighbors was associated with higher increases in the prevalence of stress during the pandemic; i.e., interquartile range (IQR) increase in % that have mutual trust among neighbors was associated with 5.9% and 18.3% decrease in prevalence of moderate stress and extreme stress during the pandemic, respectively. We postulated that social isolation might be associated with these results. Low mutual trust between neighbors could lead to weakened social ties and isolation from community, which are important risk factors for mental health problems^26^. Further, higher % with reduced physical activity due to the pandemic and lower number of sports facilities were associated with higher increases in the prevalence of stress during the pandemic. We speculate that low accessibility to sports facilities might have affected these findings. It is well known that physical activities are associated with lower levels of mental health problems^27^, and sports/recreational facilities provides better environments for recreational activities and exercise, which can alleviate stress^33,34^.

Further, local tax per person was positively associated with increase in depression; i.e., IQR increase in local tax per person (100,000 won) was associated with 5.7% increase in prevalence in depression. In Korea, there are eleven local taxes, and they are divided into province and city and district taxes. At the province level, there are four ordinary taxes (acquisition tax, registration and license tax, leisure tax, and local consumption tax) and two earmarked taxes (community resource and facility tax). At the city and district level, there are five ordinary taxes (inhabitant tax, property tax, automobile tax, local income tax, and tobacco consumption tax). Therefore, districts with higher local tax per person can be regarded as having higher socioeconomic status. We postulated that different levels of response to social distancing policies by district-level SES could be linked to a change in depression levels during and before the pandemic. Previous studies reported that mobility in cities with higher SES decreased faster than cities with lower SES following lockdown^35,36^. As the social distancing policies went on, residents in high SES districts seem to have more trouble engaging in recreational or outdoor activities^36^, which help in reducing concerns and worries^37^. In contrast, there was no association between district-level SES and increase in stress, and this result could be affected by stress resilience^38^. Because community resources that related to community resilience are generally more distributed in districts with higher SES than lower SES, stress levels in districts with high SES might have reduced compared to levels onset of pandemic. Since resilience could mediate the relationship between stress and depression^39^, it is plausible that depression in districts with high SES might also recover as time goes by; however, it should be proved on future studies.

This study has some limitations. First, there may be latent problems with regard to misclassification or recall bias due to the limitations of self-reported data. However, the KCHS was run by trained interviewers, and the quality of the KCHS was well managed systematically^21^; therefore, bias in our results would be small. Second, because we used a cross-sectional survey that did not provide individual follow-up data, causal relationships could not be established in this study. In particular, it seems more difficult to apply causal concepts, as bias in district-level characteristics can be caused by many other factors, such as geographical conditions and regional policies. Third, because of the high correlation among the regional variables as shown in the PCA result, we could not consider them simultaneously in the model. Further study should consider appropriate methods to address this issue.

## Conclusion

This study suggests that the prevalence of mental health outcomes has increased during the COVID-19 pandemic, and increases in stress and depression are more pronounced in high urbanicity districts, younger adults and high SES groups. In addition, district-level characteristics related to SES, social isolation, and physical activity were associated with changes in mental health outcome. To the best of our knowledge, this is the first and largest epidemiological study to investigate changes in mental health outcomes comparing the period of the COVID-19 pandemic (2020) to before the pandemic (2017–2019), and the associated individual and district-level characteristics using a nationwide survey in Asia. Our study might suggest the need for modifying the social distancing policy considering the individual and regional characteristics. Additionally, we can provide epidemiological evidence for establishing public health policies on the priority of resource allocation for managing mental health during the unprecedented pandemic.

## Supplementary Information

Supplementary Information.

## Data Availability

The datasets used in this study are available from the Korea Community Health Survey Data (https://chs.cdc.go.kr).
