# Supplementary material for: Individual and regional characteristics associated with changes in mental health before and during the COVID-19 pandemic in South Korea
Source: Sci Rep. 2022 Aug 19;12:14189. doi: 10.1038/s41598-022-18493-1 (PMC9390104; doi:10.1038/s41598-022-18493-1)
Supplement: Supplementary file 1 — Supplementary Information. [file 41598_2022_18493_MOESM1_ESM.pdf]

Supplementary Materials

Title. Individual and regional characteristics associated with changes in mental health before and during the COVID-19 pandemic in South Korea

Authors. Jieun Min, Dohoon Kwon, Whanhee Lee, Cinoo Kang, Chaerin Park, Seulkee Heo, Michelle L. Bell, and Ho Kim

Jieun Min and Dohoon Kwon contributed equally to this research as co-first authors.

Contents

1. Supplementary methods

2. Supplementary tables (Table S1–S6)

3. Supplementary figures (Figure S1–S3)

1. Supplementary methods

Sub-district and sub-population analyses. Based on the first stage results and previous studies presenting the close association between urbanicity and the Coronavirus Disease 2019 (COVID-19) pandemic[1], we recognized that regional urbanicity is one of the major factors that can explain the spatial heterogeneity of the first stage estimates. Thus, we divided 229 districts into three sub-districts based on population density, which has been suggested as a suitable urbanicity indicator in Korea[2]: low-density, mid-density, and high-density districts based on 33.3% and 66.7% percentile of population density. We repeated the two-stage analysis mentioned in main manuscript for each sub-district.

To identify the individual-level characteristics that affect the temporal changes in mental health outcomes, we conducted sub-population analysis. We repeated the two-stage analysis with restricting data to the subset with sex (female and male), age groups (aged 19–59 y and aged 60+ y), education level (less than college and college or higher), and household income (low and high). In addition, to check the potential association of age with education and income, double-stratification analyses were conducted by age-education and age-household income strata.

2. Supplementary tables

Table S1. Individual variables and their categorization

Variables	Categorization
**Demographic factors**	
Sex	Female / Male
Age	Aged 19–59 y / Aged 60+ y
Subjective health level	Bad / Normal / Good / Non-response
Smoking status	Never-smoker / Past-smoker / Current-smoker / Non-response
Drinking status	Never-drinker / Past-drinker / Current-drinker / Non-response
**Socioeconomic factors**	
Education level	Less than college / College or higher / Non-response
Employed	No / Yes / Non-response
Household income[a]	Low / High / Non-response
Marital status	Married / Divorced / Widowed / Separated / Never married / Non-response
Currently living alone	No / Yes
**Chronic disease history**	
Hypertension	No / Yes / Non-response
Diabetes	No / Yes / Non-response

[a]Low-household income corresponds to household income less than 3 million won (about 2,600$) per month, and high-household income corresponds to household income more than 3 million won (about 2,600$) per month.

Table S2. District-level variables and their sources

Variables	Definition	Data source
Local tax per person (100,000 won)	A local tax is an assessment by first-level local authority district (shi/do) and second-level local authority district (shi/gun/gu) in South Korea.	Korea Ministry of Public Administration and Security
% that have mutual trust among neighbors	The % that have mutual trust among neighbors was defined as the percentage of people that responded "I can trust neighbors in my town." in the Korea Community Health Survey (KCHS). This index was standardized by age based on the 2005 census.	Korea Community Health Survey
Park area per capita (m^2)	All parks prescribed by ordinance were considered.	Korea Land and Housing Corporation
# of sports facilities per 100,000 people	Sports facilities included playgrounds, stadium, golf courses, and ski places.	Korea Land and Housing Corporation
% with reduced physical activity due to the pandemic	The % with reduced physical activity due to the pandemic was defined as the percentage of people that responded "My physical activity has decreased since pandemic." in the KCHS. This index was standardized by age based on the 2005 census.	Korea Community Health Survey
% that believe in the government responses to the pandemic	The % that believe in the government responses to the pandemic was defined as the percentage of people that responded "The responsive ability to the pandemic of Korea government is very appropriate or appropriate." in likert 5 scale questionnaire of the KCHS. This index was standardized by age based on the 2005 census.	Korea Community Health Survey
# of COVID-19 confirmed cases	We used cumulative number of COVID-19 confirmed cases until 31 October, 2020.	Korea Statistical Information Service

Table S3. Top 20 districts with low prevalence of moderate stress, extreme stress, and depression before the outbreak of the COVID-19 pandemic (2017–2019) and the changes in prevalence of mental health outcomes before (2017–2019) and during (2020) the pandemic[a]

Moderate stress		Extreme stress		Depression	
District	Change in prevalence (%)	District	Change in prevalence (%)	District	Change in prevalence (%)
Yeongkeok-gun, GB	5.6	Yeongdeok-gun, GB	0.5	Ulleung-gun, GB	0.0
Seongju-gun, GB	1.6	Ulleung-gun, GB	-0.4	Yeongdeok-gun, GB	2.5
Sangju-shi, GB	4.0	Sangju-shi, GB	1.0	Sangju-shi, GB	0.8
Sacheon-shi, GN	15.9	Sacheon-shi, GN	3.7	Gurye-gun, JN	-0.7
Jangsu-gun, JB	-1.8	Goseong-gun, GN	-0.8	Changnyeong-gun, GN	-0.5
Sunchang-gun, JB	3.5	Yongsan-gu, Seoul	2.3	Ulju-gun, Ulsan	-0.7
Sancheong-gun, GN	1.4	Jangsu-gun, JB	1.6	Yecheon-gun, GB	3.6
Ulleung-gun, GB	3.6	Muan-gun, JN	-0.3	Muan-gun, JN	0.2
Yeongdong-gun, CB	.2	Yangyang-gun, GW	0.1	Goseong-gun, GW	0.9
Changnyeong-gun, GN	-3.0	Seo-gu, Daegu	0.6	Yeoncheon-gun, GG	2.8
Yeoncheon-gun, GG	5.3	Changnyeong-gun, GN	-1.1	Gunwi-gun, GB	3.8
Sokcho-shi, GW	-2.2	Hampyeong-gun, JN	-0.9	Yangyang-gun, GW	0.2
Goseong-gun, GN	-3.0	Cheorwon-gun, GW	1.4	Uljin-gun, GB	0.5
Uljin-gun, GB	-2.4	Geoje-shi, GN	0.8	Seo-gu, Daegu	1.2
Goheung-gun, JN	-1.7	Yeongyang-gun, GB	1.8	Goseong-gun, GN	1.1
Yongsan-gu, Seoul	4.7	Seonju-gun, GB	1.6	Seongju-gun, GB	0.2
Jinan-gun, JB	3.2	Sokcho-shi, GW	-1.0	Boseong-gun, JN	-0.7
Hadong-gun, GN	7.1	Hamyang-gun, GN	4.3	Hampyeong-gun, JN	-0.8
Gurye-gun, JN	-5.5	Goseong-gun, GW	-1.1	Buk-gu, Busan	0.5
Wando-gun, JN	3.8	Buan-gun, JB	1.8	Sokcho-shi, GW	-1.5

CB, chungcheongbuk-do; CN, chungcheongnam-do; GB, gyungsangbuk-do; GG, gyunggi-do; GN, gyungsangnam-do; GW, gangwon-do; JB, jeollabuk-do; JN, jeollanam-do
[a]The mental health prevalence was standardized by age based on the 2005 census.

Table S4. Top 20 districts with high prevalence of moderate stress, extreme stress, and depression before the outbreak of the COVID-19 pandemic (2017–2019) and the changes in prevalence of mental health outcomes before (2017–2019) and during (2020) the pandemic[a]

Moderate stress		Extreme stress		Depression	
District	Change in prevalence (%)	District	Change in prevalence (%)	District	Change in prevalence (%)
Michuhol-gu, Incheon	2.0	Muju-gun, JB	-1.5	Bupyeong-gu, Incheon	-2.4
Bupyeong-gu, Incheon	3.4	Michuhol-gu, Incheon	0.4	Dong-gu, Incheon	-2.5
Seongdong-gu, Seoul	0.2	Gangnam-gu, Seoul	-0.7	Ansan-shi, GG	-0.3
Cheonan-shi, CN	0.0	Bupyeong-gu, Incheon	0.8	Namyangju-shi, GG	-2.6
Seosan-shi, CN	-3.2	Bonghwa-gun, GB	-1.9	Seosan-shi, CN	-3.4
Ansan-shi, GG	1.2	Gangseo-gu, Seoul	-0.6	Jecheon-shi, CB	-2.4
Gangseo-gu, Seoul	-0.0	Wonju-shi, GW	0.1	Michuhol-gu, Incheon	-0.8
Hongseong-gun, CN	-1.2	Gongju-shi, CN	-1.4	Jeonju-shi, JB	-2.2
Icheon-shi, GG	1.3	Yeongwol-gun, GW	-3.2	Gochang-gun, JB	-6.2
Gokseong-gun, JN	-5.7	Geumcheon-gu, Seoul	-0.2	Gimje-shi, JB	-2.9
Dongdaemun-gu, Seoul	-4.5	Seosan-shi, CN	-1.7	Yeoju-shi, GG	-2.9
Goyang-shi, GG	0.5	Ongjin-gun, Incheon	-1.3	Yeonje-gu, Busan	1.3
Seocho-gu, Seoul	-1.4	Seongbuk-gu, Seoul	-0.3	Jung-gu, Busan	-0.1
Gwangmyeong-shi, GG	-0.1	Gimcheon-shi, GB	-2.4	Osan-shi, GG	-2.1
Jungnang-gu, Seoul	4.7	Bucheon-shi, GG	-0.1	Gangneung-shi, GW	-1.4
Dong-gu, Gwangju	-0.6	Gochang-gun, JB	-1.6	Gyeongsan-shi, GB	1.9
Bucheon-shi, GG	1.8	Sasang-gu, Busan	-2.3	Jungnang-gu, Seoul	0.0
Jung-gu, Ulsan	-5.1	Jongno-gu, Seoul	-1.8	Uiwang-shi, GG	-0.7
Seongbuk-gu, Seoul	3.9	Gokseong-gun, JN	-2.9	Eumseong-gun, CB	0.9
Gochang-gun, JB	-9.4	Uiwang-shi, GG	0.7	Andong-shi, GB	-2.1

CB, chungcheongbuk-do; CN, chungcheongnam-do; GB, gyungsangbuk-do; GG, gyunggi-do; GN, gyungsangnam-do; GW, gangwon-do; JB, jeollabuk-do; JN, jeollanam-do

[a]The mental health prevalence was standardized by age based on the 2005 census.

Table S5. Associations between district-level characteristics and change in mental health comparing the time period of the COVID-19 pandemic (2020) to the time period before the pandemic (2017–2019), by population density

	Percentile change in OR (95% CI)[a]		
	Low population-density[b]	Mid population-density[b]	High population-density[b]
**Moderate stress**			
Local tax per person (100,000 won)	-6.6 (-19.8, 8.7)	0.5 (-3.2, 4.3)	0.7 (-1.6, 3.1)
% that have mutual trust among neighbors	-5.8 (-18.7, 9.0)	-10.3 (-20.3, 1.0)	13.5 (-0.6, 29.6)
Park area per capita (m^2)	-8.9 (-16.5, -0.5)*	-0.7 (-5.3, 4.3)	-0.1 (-7.0, 7.4)
# of sports facilities per 100,000 people	-8.9 (-15.6, -1.7)*	-0.1 (-12.7, 14.4)	1.3 (-40.5, 72.6)
% with reduced physical activity due to the pandemic	8.0 (-2.3, 19.4)	12.2 (2.3, 23.1)*	16.4 (0.6, 34.7)*
% that believe in the government responses to the pandemic	-2.4 (-11.9, 8.1)	6.2 (-3.7, 17.2)	4.0 (-8.7, 18.6)
# of COVID-19 confirmed cases	-5.8 (-34.8, 36.2)	0.4 (-14.3, 17.7)	-1.0 (-14.9, 15.2)
**Extreme stress**			
Local tax per person (100,000 won)	-15.4 (-41.5, 22.3)	6.6 (-1.9, 15.9)	-1.4 (-5.6, 3.1)
% that have mutual trust among neighbors	-22.8 (-46.5, 11.4)	-18.0 (-37.9, 8.2)	15.9 (-11.4, 51.4)
Park area per capita (m^2)	-3.1 (-22.8, 21.6)	6.1 (-4.9, 18.4)	-1.1 (-11.5, 15.5)
# of sports facilities per 100,000 people	-11.5 (-26.7, 6.9)	9.9 (-19.7, 50.2)	-29.0 (-73.4,89.2)
% with reduced physical activity due to the pandemic	8.3 (-16.5, 40.5)	24.0 (-0.7, 54.8)	28.4 (-4.1, 72.0)
% that believe in the government responses to the pandemic	-5.1 (-26.9, 23.1)	5.2 (-16.3, 32.2)	-6.1 (-27.3, 21.3)
# of COVID-19 confirmed cases	-27.3 (-69.8, 75.0)	2.1 (-29.4, 47.7)	-12.5 (-35.4, 18.5)
**Depression**			
Local tax per person (100,000 won)	-0.7 (-27.0, 35.1)	5.9 (-2.0, 14.5)	6.3 (1.6, 11.2)*
% that have mutual trust among neighbors	-6.5 (-30.9, 26.4)	-10.1 (-31.6, 18.3)	21.4 (-4.3, 54.0)
Park area per capita (m^2)	8.9 (-9.4, 30.9)	-0.2 (-9.9, 10.6)	-3.2 (-14.1, 9.2)
# of sports facilities per 100,000 people	10.1 (-6.1, 29.0)	6.8 (-19.0, 40.8)	-21.3 (-68.8, 98.6)
% with reduced physical activity due to the pandemic	-1.3 (-20.2, 22.1)	2.1 (-18.3, 27.6)	2.5 (-21.8, 34.3)
% that believe in the government responses to the pandemic	-8.2 (-25.6, 13.3)	-19.0 (-34.5, 0.2)	-6.7 (-26.1, 17.9)
# of COVID-19 confirmed cases	-6.2 (-56.6, 102.5)	29.2 (-7.1, 79.8)	-1.2 (-24.8, 29.7)

CI, confidence interval; OR, odds ratio

[a]Percentile change in ORs and 95% CIs were calculated from meta-regression after adjusting for longitude and latitude of each district. Results were expressed as percentile change in OR of mental health for interquartile range (IQR) increase of the district-level variables.

[b]Three sub-districts (low-density, mid-density, and high-density districts) were divided based on 33.3% and 66.7% percentile of population density.

*$P<0.05$

Table S6. Associations between district-level characteristics and change in mental health comparing the time period of the COVID-19 pandemic (2020) to the time period before the pandemic (2017–2019), by sex and age

	Percentile change in OR (95% CI)[a]			
	Female	Male	Aged 19–59 y	Aged 60+ y
**Moderate stress**				
Local tax per person (100,000 won)	0.5 (-1.8, 2.7)	1.2 (-1.2, 3.7)	0.2 (-1.9, 2.4)	2.0 (-1.0, 5.0)
% that have mutual trust among neighbors	-7.5 (-12.7, -1.9)*	-3.3 (-9.8, 3.5)	-3.0 (-8.7, 3.2)	-6.0 (-12.7, 1.2)
Park area per capita (m^2)	-4.5 (-8.3, -0.9)*	-1.6 (-5.8, 2.8)	-2.8 (-6.4, 0.9)	-3.1 (-7.7, 1.6)
# of sports facilities per 100,000 people	-9.3 (-13.4, -4.9)*	-4.5 (-9.9, 1.2)	-5.1 (-10.0, 0.0)	-8.0 (-13.1, -2.5)*
% with reduced physical activity due to the pandemic	12.4 (6.7, 18.4)*	7.6 (1.1, 14.5)*	9.4 (3.4, 15.7)*	8.5 (1.6, 15.7)*
% that believe in the government responses to the pandemic	0.5 (-5.8, 7.2)	-1.2 (-8.4, 6.4)	-1.1 (-7.5, 5.7)	3.8 (-4.1, 12.4)
# of COVID-19 confirmed cases	7.9 (-2.1, 18.9)	3.1 (-7.8, 15.3)	3.9 (-5.7, 14.4)	4.3 (-8.3, 18.5)
**Extreme stress**				
Local tax per person (100,000 won)	2.7 (-2.2, 7.8)	2.7 (-9.1, 16.1)	2.5 (-7.9, 14.2)	9.8 (-13.0, 38.5)
% that have mutual trust among neighbors	-17.8 (-28.0, -6.0)*	-46.9 (-61.5, -26.7)*	-31.2 (-48.5, -8.1)*	0.4 (-45.2, 84.1)
Park area per capita (m^2)	-1.1 (-9.0, 7.5)	-20.9 (-35.9, -2.4)*	-13.4 (-28.1, 4.1)	11.5 (-24.8, 65.3)
# of sports facilities per 100,000 people	-13.1 (-22.0, -3.2)*	-26.5 (-44.0, -3.4)*	-21.5 (-38.4, 0.0)	3.9 (-36.8, 70.7)
% with reduced physical activity due to the pandemic	30.2 (14.5, 48.0)*	94.0 (45.2, 159.2)*	56.7 (20.8, 103.4)*	87.3 (9.3, 220.9)*
% that believe in the government responses to the pandemic	-13.3 (-25.3, 0.5)	37.2 (-4.2, 96.6)	40.6 (2.4, 93.1)*	-13.5 (-55.3, 67.3)
# of COVID-19 confirmed cases	19.2 (-4.8, 49.2)	10.6 (-36.6, 92.9)	8.6 (-33.4, 77.0)	-18.1 (-70.9, 131.0)
**Depression**				
Local tax per person (100,000 won)	7.3 (2.6, 12.2)*	4.1 (-1.7, 10.2)	6.1 (1.6, 10.9)*	4.4 (-2.5, 11.8)
% that have mutual trust among neighbors	0.2 (-10.4, 12.0)	-7.9 (-21.0, 7.3)	3.2 (-8.9, 16.8)	-11.1 (-24.6, 4.9)
Park area per capita (m^2)	-0.1 (-6.8, 7.1)	0.1 (-8.9, 10.1)	-1.3 (-8.3, 6.3)	1.1 (-9.1, 12.5)
# of sports facilities per 100,000 people	4.3 (-4.6, 14.1)	-0.1 (-11.7, 13.1)	6.2 (-4.1, 17.6)	4.7 (-8.2, 19.6)
% with reduced physical activity due to the pandemic	0.3 (-9.4, 11.1)	10.1 (-4.6, 27.1)	0.3 (-10.8, 12.8)	14.1 (-1.8, 32.7)
% that believe in the government responses to the pandemic	-8.8 (-19.3, 2.9)	-8.7 (-22.9, 8.0)	-11.5 (-22.6, 1.3)	12.3 (-6.3, 34.8)
# of COVID-19 confirmed cases	11.2 (-7.3, 33.4)	5.9 (-17.3, 35.7)	9.6 (-9.3, 32.5)	-0.6 (-25.3, 32.3)

CI, confidence interval; OR, odds ratio

[a]Percentile change in ORs and 95% CIs were calculated from meta-regression after adjusting for longitude and latitude of each district. Results were expressed as percentile change in OR of mental health for interquartile range (IQR) increase of the district-level variables.

*P<0.05

Table S7. Associations between district-level characteristics and change in mental health comparing the time period of the COVID-19 pandemic (2020) to the time period before the pandemic (2017–2019), by education level and household income

	Percentile change in OR (95% CI)[a]			
	Less than college	College or higher	Low-income[b]	High-income[b]
**Moderate stress**				
Local tax per person (100,000 won)	2.1 (-0.4, 4.6)	0.2 (-2.4, 2.8)	0.8 (-2.0, 3.8)	0.6 (-1.7, 3.0)
% that have mutual trust among neighbors	-7.0 (-12.2, -1.5)*	-1.0 (-8.7, 7.3)	-5.2 (-11.4, 1.3)	-4.5 (-10.9, 2.4)
Park area per capita (m^2)	-3.6 (-7.3, 0.3)	-1.7 (-6.1, 3.0)	-2.3 (-6.6, 2.3)	-3.4 (-7.3, 0.7)
# of sports facilities per 100,000 people	-7.5 (-11.7, -3.1)*	-3.8 (-10.4, 3.4)	-6.8 (-11.6, -1.7)*	-6.4 (-11.9, -0.7)*
% with reduced physical activity due to the pandemic	8.5 (3.0, 14.2)*	11.9 (3.7, 20.7)*	11.2 (4.8, 18.0)*	8.8 (2.0, 16.1)*
% that believe in the government responses to the pandemic	2.1 (-4.1, 8.8)	-4.8 (-12.8, 3.9)	-1.5 (-8.4, 6.0)	1.0 (-6.4, 8.9)
# of COVID-19 confirmed cases	7.5 (-2.7, 18.8)	2.4 (-9.2, 15.6)	8.6 (-3.4, 21.9)	1.5 (-8.7, 12.9)
**Extreme stress**				
Local tax per person (100,000 won)	-4.7 (-12.5, 3.7)	2.9 (-10.3, 18.0)	-0.2 (-6.3, 6.4)	7.2 (-8.0, 24.8)
% that have mutual trust among neighbors	-17.9 (-34.3, 2.6)	-48.9 (-64.7, -26.1)*	-19.4 (-30.8, -6.2)*	-50.0 (-66.7, -24.9)*
Park area per capita (m^2)	3.2 (-10.8, 19.4)	-13.8 (-32.1, 9.3)	-0.2 (-9.9, 10.5)	-8.5 (-29.7, 19.1)
# of sports facilities per 100,000 people	-13.1 (-27.4, 4.1)	-35.0 (-52.6, -10.8)*	-12.1 (-22.2, -0.7)*	-31.3 (-51.2, -3.4)*
% with reduced physical activity due to the pandemic	8.1 (-11.9, 32.7)	149.5 (79.0, 247.7)*	21.1 (5.3, 12.9)*	128.3 (58.4, 229.0)*
% that believe in the government responses to the pandemic	2.5 (-19.9, 31.2)	28.1 (-15.7, 94.7)	-4.7 (-19.6, 12.9)	52.6 (-3.0, 139.9)
# of COVID-19 confirmed cases	-9.8 (-38.6, 32.4)	45.7 (-21.8, 171.4)	5.6 (-19.3, 38.2)	36.7 (-31.2, 171.8)
**Depression**				
Local tax per person (100,000 won)	5.9 (-0.8, 13.0)	4.4 (-9.3, 20.2)	7.6 (1.7, 13.8)*	5.9 (-2.6, 15.1)
% that have mutual trust among neighbors	-12.1 (-24.9, 2.8)	74.0 (18.3, 156.0)*	2.3 (-10.3, 16.6)	-24.0 (-39.7, -4.2)*
Park area per capita (m^2)	3.1 (-6.9, 14.3)	-2.4 (-23.5, 24.5)	2.6 (-5.9, 11.9)	-11.1 (-23.0, 2.7)
# of sports facilities per 100,000 people	5.0 (-7.5, 19.2)	20.1 (-13.0, 65.8)	11.3 (0.4, 23.4)*	-15.3 (-30.3, 2.9)
% with reduced physical activity due to the pandemic	16.1 (0.6, 33.9)*	-46.0 (-62.0, -23.2)*	-4.2 (-15.0, 8.0)	30.1 (5.2, 60.8)*
% that believe in the government responses to the pandemic	10.5 (-7.0, 31.4)	-53.6 (-69.3, -30.0)*	-10.8 (-22.8, 3.0)	-22.5 (-39.7, -0.3)*
# of COVID-19 confirmed cases	6.8 (-18.3, 39.4)	-6.2 (-50.2, 76.5)	2.6 (-17.9, 28.2)	19.0 (-18.0, 72.8)

CI, confidence interval; OR, odds ratio

[a]Change in ORs and 95% CIs were calculated from meta-regression after adjusting for longitude and latitude of each district. Results were expressed as percentile change in OR of mental health for interquartile range (IQR) increase of the district-level variables.

[b]Low-income corresponds to household income less than 3 million won (about 2600$) per month, and high-income corresponds to household income more than 3 million won per month.

*$P<0.05$

Table S8. Associations between district-level characteristics and standardized rate change of mental health prevalence comparing the time period of the COVID-19 pandemic (2020) to the time period before the pandemic (2017–2019)

	Percentile change in β (95% CI)[a]
**Moderate stress**	
Local tax per person (100,000 won)	0.05 (-0.25, 0.36)
% that have mutual trust among neighbors	-0.62 (-1.41, 0.16)
Park area per capita (m^2)	-0.63 (-1.15, -0.12)*
# of sports facilities per 100,000 people	-1.05 (-1.69, -0.41)*
% with reduced physical activity due to the pandemic	1.36 (0.67, 2.05)*
% that believe in the government responses to the pandemic	-0.62 (-1.48, 0.24)
# of COVID-19 confirmed cases	0.73 (-0.62, 2.08)
**Extreme stress**	
Local tax per person (100,000 won)	-0.01 (-0.11, 0.10)
% that have mutual trust among neighbors	-0.10 (-0.38, 0.18)
Park area per capita (m^2)	-0.01 (-0.19, 0.18)
# of sports facilities per 100,000 people	0.02 (-0.21, 0.25)
% with reduced physical activity due to the pandemic	0.36 (0.11, 0.60)*
% that believe in the government responses to the pandemic	-0.08 (-0.38, 0.23)
# of COVID-19 confirmed cases	0.00 (-0.48, 0.48)
**Depression**	
Local tax per person (100,000 won)	0.09 (-0.06, 0.23)
% that have mutual trust among neighbors	0.01 (-0.36, 0.39)
Park area per capita (m^2)	-0.06 (-0.31, 0.18)
# of sports facilities per 100,000 people	0.15 (-0.16, 0.47)
% with reduced physical activity due to the pandemic	0.04 (-0.30, 0.38)
% that believe in the government responses to the pandemic	-0.29 (-0.70, 0.11)
# of COVID-19 confirmed cases	0.29 (-0.35, 0.94)

CI, confidence interval

[a]Coefficients (β) and 95% CIs were calculated from multiple linear regression after adjusting for longitude and latitude of each district. Results were expressed as regression coefficients for interquartile range (IQR) increase of the district-level variables.

*P <0.05

3. Supplementary figures

Figure S1. Score plot and loading plot of the principal component analysis (PCA)
Believing in government responses, % that believe in government responses to the pandemic; COVID-19 confirmed cases, number of COVID-19 confirmed cases; Local tax, local tax per person; Mutual trust, % that have mutual trust among neighbors; Park area, park area per capita; Reduced physical activity, % with reduced physical activity due to pandemic; Sports facilities, number of sports facilities per 100,000 people

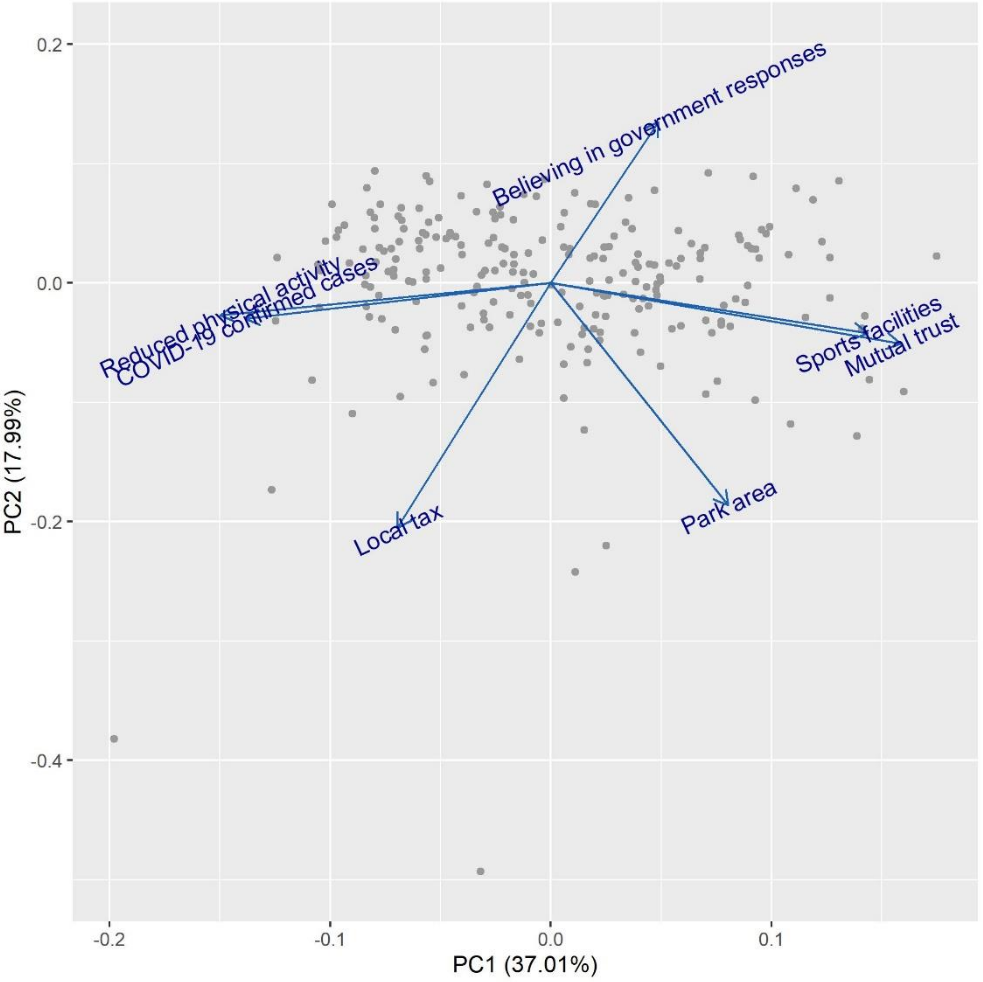

Figure S2. Geographical distributions of low population-density, mid population-density, and high population-density districts and odds ratio (OR) of mental health by the outbreak of the COVID-19 pandemic. (a) Population density; (b) OR for moderate stress; (c) OR for extreme stress; and (d) OR for depression. ORs reflect a comparison of the time period of the pandemic to the period before the pandemic and were calculated from pooled-analysis after adjusting for time trend and individual characteristics. The shapefile for this map was obtained from the National Geographic Information Institute (http://data.nsdi.go.kr/dataset/20180927ds0058) which is publicly available and the figure was created using R software (version 4.1.0).

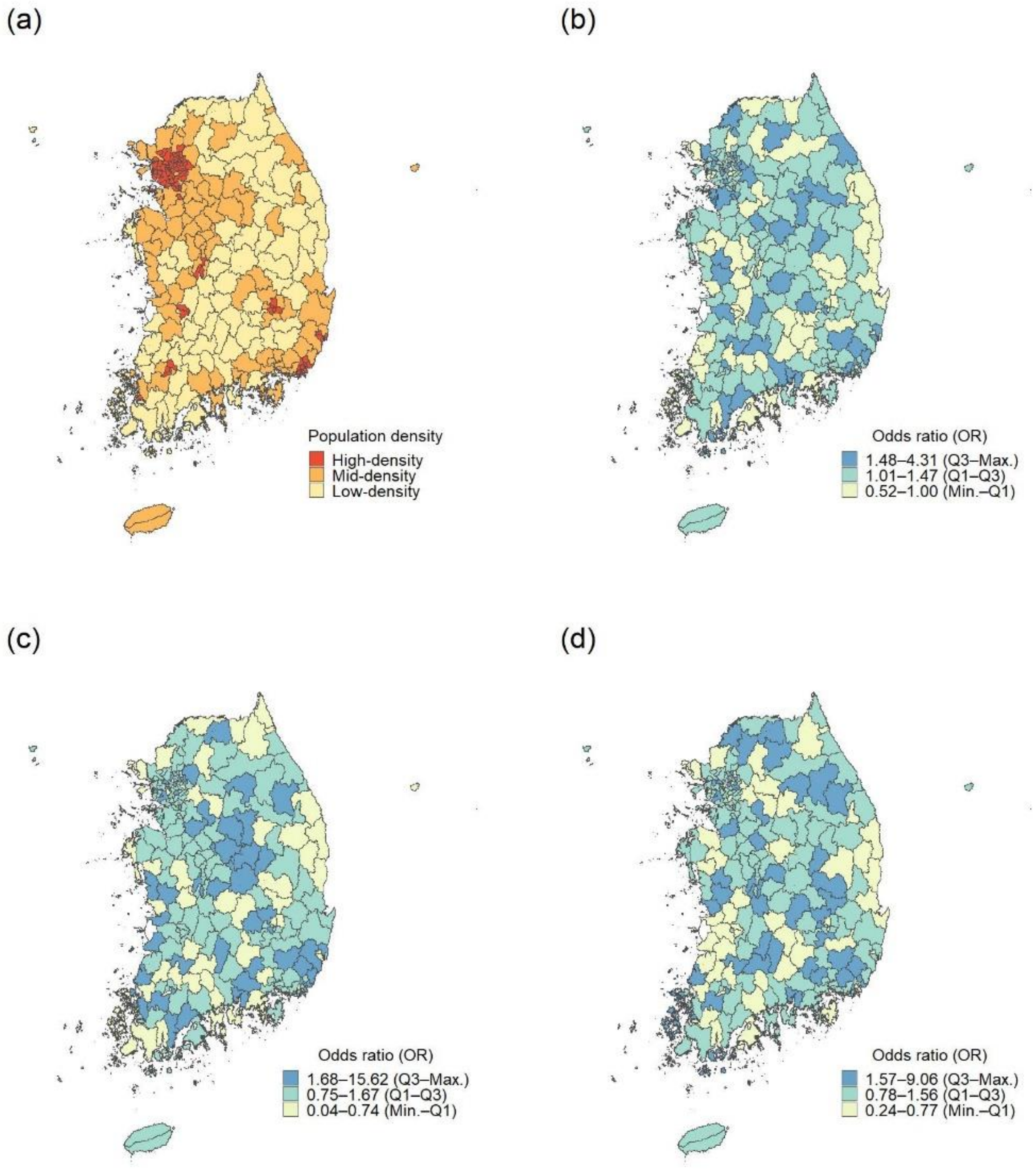

Figure S3. Time trend adjusted odds ratio (OR) of mental health outcomes by the outbreak of the COVID-19 pandemic according to double-stratification sub-population. (a) Moderate stress; (b) Extreme stress; and (c) Depression. ORs and 95% CIs were calculated from pooled-analysis after adjusting for time trend and individual characteristics. Low-household income corresponds to household income less than 3 million won (about 2,600$) per month, and high-household income corresponds to household income more than 3 million won (about 2,600$) per month.

CI, confidence interval

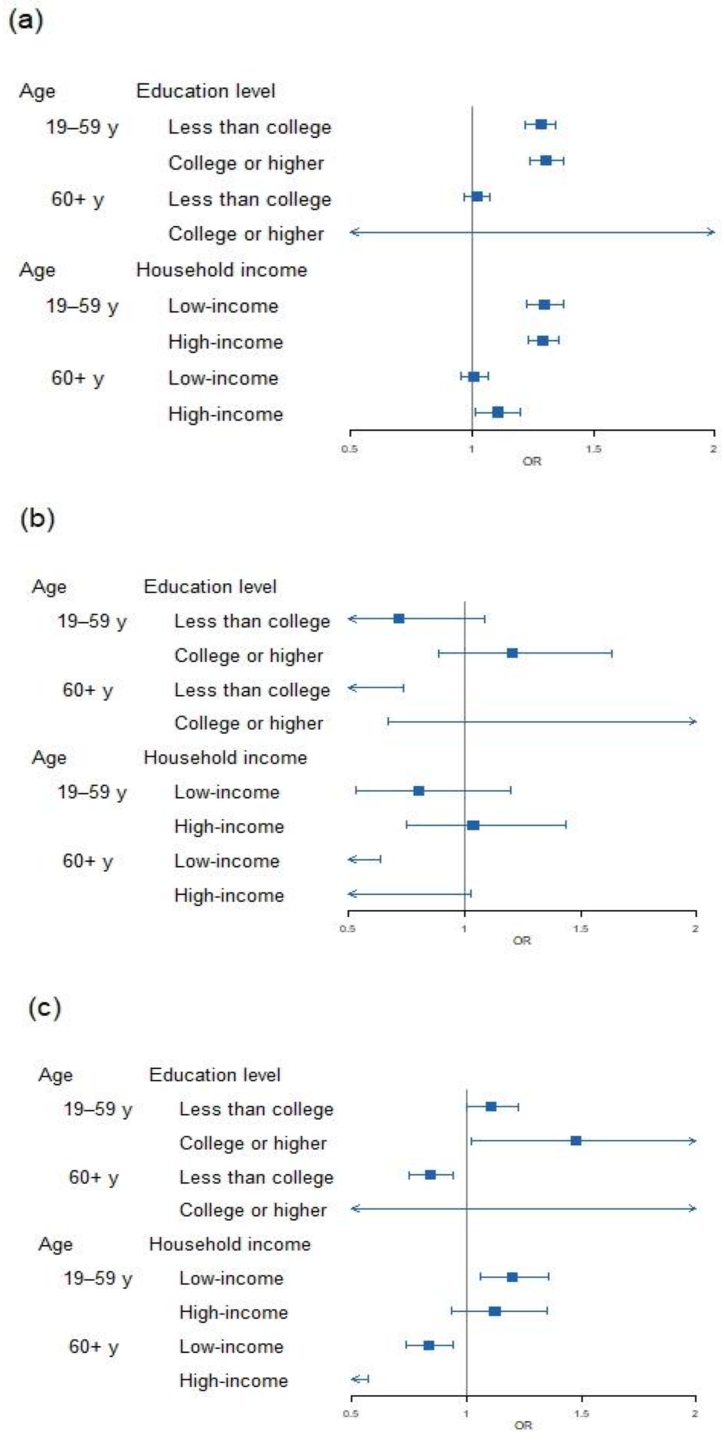

References

[1] Lee, W. *et al.* Urban environments and COVID-19 in three Eastern states of the United States. *Sci Total Environ* **779**, 146334. https://doi.org/10.1016/j.scitotenv.2021.146334 (2021).

[2] Lee, W. *et al.* Effects of urbanization on vulnerability to heat-related mortality in urban and rural areas in South Korea: a nationwide district-level time-series study. *Int J Epidemiol*. https://doi.org/10.1093/ije/dyab148 (2021).
